# The Comparison of QTc Dispersion Between Renal Transplant Recipients and Healthy Individuals

**DOI:** 10.7759/cureus.32458

**Published:** 2022-12-12

**Authors:** Musa Ilker Durak, Beyza Algul Durak

**Affiliations:** 1 Cardiology, Ankara Bilkent City Hospital, Ankara, TUR; 2 Nephrology, Ankara Bilkent City Hospital, Ankara, TUR

**Keywords:** repolarization, arrhythmia, renal transplantation, corrected qt dispersion, qt dispersion

## Abstract

Introduction

Cardiovascular diseases are the most common cause of death in patients with end-stage renal failure. The increase in QTc interval time and QT dispersion increases the risk of cardiac arrhythmia and mortality. In our study, QT and QTc dispersions (QTcd) of patients who underwent renal transplantation were compared with normal healthy individuals.

Methods

Electrocardiograms (ECGs) of 80 renal transplant recipients and 70 healthy individuals were taken. QTc dispersion was calculated by using the longest and the shortest QT interval and QT dispersion and Bazett's formula.

Results

When the groups were compared, similar QT dispersion and QTc dispersion were observed (control group and renal transplant recipient patients: 35 ± 17 ms and 36 ± 16 ms and 52 ± 18 ms and 54 ± 22 ms, respectively).

Conclusion

No statistical difference was observed between QT and QTc dispersions of renal transplant patients compared to healthy individuals. This result shows that the increase in QT dispersion and pathophysiological mechanisms observed frequently in uremic patients can be reversed by renal transplantation.

## Introduction

Many changes can be observed in the electrocardiogram (ECG) of patients undergoing hemodialysis treatment. ECG changes and arrhythmias may be related to both renal failure and hemodialysis [1]. The cardiovascular manifestations in these patients are congestive heart failure and coronary artery disease; however, sudden cardiac death due to hyperkalemia might be seen.

Renal transplantation is an effective treatment option that reduces the rate of mortality and morbidity due to renal failure in patients with end-stage kidney disease [2]. On the other hand, cardiovascular events can be observed in the follow-up of patients undergoing renal transplantation due to pretransplantation processes, the direct effect of immunosuppressive therapy, or other risk factors [3].

Basically, QT dispersion is the difference between the maximum and minimum QT intervals in 12-lead ECG, indicating heterogeneity in repolarization [4]. There are studies showing that the increase in QT dispersion can be used as an early mortality predictor in diseases such as acute myocardial infarction, long QT syndromes, chronic heart failure, or dilated cardiomyopathy [5,6].

The aim of our study was to compare the QT dispersion of the patients undergoing renal transplantation and the healthy control group.

## Materials and methods

In the study, 80 renal transplant patients (47 females and 33 males, 42.2 ± 14.5 years old) were included. The exclusion criteria were a history of coronary artery disease or cardiomyopathy, having a bundle branch block, ischemic changes and arrhythmia on ECG, a history of antiarrhythmic drug use in the first three months following transplantation, and a creatinine value of ≥2.5 mg/dl. Ethics committee approval was obtained prior to the study from Ankara Bilkent City Hospital institutional review board (approval number: E2-22-1195-2), and the Helsinki Declaration principles were observed.

For the transplantation group, the etiologic factors were as follows: 30 patients had glomerulonephritis, eight had amyloidosis, 17 had tubulointerstitial nephritis, 14 had nephrosclerosis, and the etiology of 11 patients was unknown. The mean time after transplantation was 42.3 ± 27.3 months. The control group consisted of 70 healthy individuals who applied to the hospital for control purposes and did not have any disease history.

The 12-lead ECGs of all participants at 25 mm/second and 10 mV/mm were taken in the supine position after 10 minutes of rest. Five cardiac cycles were evaluated and averaged. The part from the beginning of the Q wave to the end of the T wave was measured, and the QT interval was calculated in milliseconds. Derivations where the T wave could not be clearly observed were not analyzed. QTc interval was calculated using Bazett's formula (QTc = measured QT interval {seconds} / √R-R interval {seconds}) [7]. At least nine derivations of the ECG of the patients with calculated QTc interval were examined for QTc dispersion (QTcd). QTcd was calculated by calculating the difference between the longest QTc interval and the shortest QTc interval. QTcd time over 50 ms was considered abnormal.

Serum magnesium, calcium, creatinine, sodium, potassium, and other biochemical parameters were measured by means of a computerized autoanalyzer (Hitachi 717, Boehringer Mannheim, Germany).

Statistical analysis

Continuous variables are expressed as mean ± SD. Comparisons were made using Student's t-test, Mann-Whitney U tests, and chi-square test where appropriate. Simple regression analysis was used to examine the correlations between QT and QTc dispersions and electrolytes. P < 0.05 was considered statistically significant.

## Results

In this study, 80 renal transplant recipients (47 female and 33 male) and 70 healthy individuals (44 male and 26 female) were included. The basic characteristics are presented in Table 1.

**Table 1:** Baseline characteristics

Characteristics	Numbers
Sex (%)	
Female	47 (58.75)
Male	33 (41.25)
Age (mean ± SD) (years)	42.2 ± 14.5
Etiology (%)	
Glomerulonephritis	30 (37.5)
Tubulointerstitial nephritis	17 (21.25)
Nephrosclerosis	14 (17.5)
Amyloidosis	8 (10)
Unknown	11 (13.75)

Renal transplant recipients had similar calcium, magnesium, and phosphorus levels compared to the control group. Serum creatinine levels were significantly higher in renal transplant recipients compared to the control group (1.3 ± 0.7 and 0.7 ± 0.3 mg/dl; p < 0.01). Serum potassium level was significantly higher in the renal transplant recipient group (4.6 ± 0.5 and 4.1 ± 0.5 mEq/l; p < 0.01). Similar QT dispersion and QTc dispersion were observed when the groups were compared (Table 2) (control group and renal transplant recipient patients: 35 ± 17 ms and 36 ± 16 ms and 52 ± 18 ms and 54 ± 22 ms, respectively).

**Table 2:** Laboratory and ECG characteristics of the control group and renal transplant recipients (mean ± SD) NS: not significant; ECG: electrocardiogram

	Renal transplant recipients	Control group	P value
Age, years	42.2 ± 14.5	40.1 ± 13.7	NS
Creatinine, mg/dl	1.3 ± 0.7	0.7 ± 0.3	P < 0.01
QT dispersion, ms	36 ± 16	35 ± 17	NS
QTc dispersion, ms	54 ± 22	52 ± 18	NS
K, mEq/l	4.6 ± 0.5	4.1 ± 0.5	P < 0.01
Ca, mg/dl	9.2 ± 0.5	9.0 ± 0.3	NS
P, mg/dl	3.4 ± 0.6	3.1 ± 0.7	NS
Mg, mg/dl	1.8 ± 0.3	1.8 ± 0.3	NS

## Discussion

In our study, QT and QTc dispersions were found to be similar in renal transplant recipients compared to the healthy control group.

Patients with chronic kidney disease are in the high-risk group in terms of coronary artery disease, peripheral artery disease, and congestive heart failure. With the effect of uremia and increased volume and sympathetic activity and ventricular dilatation, hypertrophy can be observed, and cardiac arrhythmias can be observed due to metabolic factors.

Prolonged QT and QT dispersion may be associated with various cardiac diseases and may trigger severe ventricular arrhythmias [8]. Studies have shown that QT dispersion increases in patients with end-stage renal failure [9]. It has been shown that QT interval prolongation in hemodialysis patients is higher than in the predialysis-stage patient group and may cause severe arrhythmia and sudden cardiac death [10].

Following renal transplantation, cardiac functions improve as the uremic condition improves; electrolyte anomalies and autonomic dysfunction also improve [11]. In a study, it has been shown that the degree of left ventricular hypertrophy and the risk of coronary artery disease decreased following renal transplantation [12,13]. Following renal transplantation, inflammation markers such as C-reactive protein, tumor necrosis factor-alpha (TNF-α), and interleukin 6 have been shown to decrease after transplantation [14]. Although the mechanism is not clear, the inflammatory process and oxidative stress may decrease with renal transplantation. It is highly probable according to the results of our study that QT dispersion can also improve with the regression of the current pathological mechanisms [15,16]. To define the pathogenesis of the decrease of QT and QTc dispersion, designed studies are needed.

Our study had several important limitations. Due to the retrospective nature of the study, a detailed analysis of the factors that may affect QT could not be done. The blood level of renal transplant recipients that took immunosuppressive drugs at the time of ECG was unknown. Because some immunosuppressive drugs have been shown to be a factor in changing the level of potassium, a decrease in QT dispersion cannot be ruled out.

## Conclusions

QT dispersion and QTc dispersion time of renal transplant recipients seem to be similar compared to healthy individuals; the data available suggest that the decrease in electrolyte, acid-base balance, left ventricular hypertrophy, and inflammation caused by renal transplantation possibly reverses the increase in QT dispersion observed in uremic patients.
